# Improving pregnant women’s knowledge on danger signs and birth preparedness practices using an interactive mobile messaging alert system in Dodoma region, Tanzania: a controlled quasi experimental study

**DOI:** 10.1186/s12978-019-0838-y

**Published:** 2019-12-12

**Authors:** Theresia J. Masoi, Stephen M. Kibusi

**Affiliations:** 1 Department of Nursing and midwifery, College of Health Sciences, the University of Dodoma, P.O. Box 259, Dodoma, Tanzania; 2 Department of Public Health, College of Health Scinces, the University of Dodoma, Dodoma, Tanzania

**Keywords:** Messaging system, Obstetric and newborn danger signs, Birth preparedness, Antenatal care, Maternal and child health, Health information systems, And mHealth

## Abstract

**Background:**

Unacceptably high maternal and perinatal mortality remain a major challenge in many low income countries. Early detection and management of danger signs through improved access to maternal services is highly needed for better maternal and infant outcomes. The aim of this study was to test the effectiveness of an interactive mobile messaging alert system on improving knowledge on danger signs, birth preparedness and complication readiness practices among pregnant women in Dodoma region, Tanzania.

**Methods:**

**A** controlled quasi experimental study of 450 randomly selected pregnant women attending antenatal care was carried in Dodoma municipal. Participants were recruited at less than 20 weeks of gestation during the first visit where 150 were assigned to the intervention and 300 to the control group. The intervention groups was enrolled in an interactive mobile messaging system and received health education messages and were also able to send and receive individualized responses on a need basis. The control group continued receiving usual antenatal care services offered at the ANC centers. Pregnant women were followed from their initial visit to the point of delivery. Level of knowledge on danger signs and birth preparedness were assessed at baseline and a post test was again given after delivery for both groups. Analyses of covariance, linear regression were employed to test the effectiveness of the intervention.

**Results:**

The mean age of participants was 25.6 years ranging from 16 to 48 years. There was significant mean scores differences for both knowleadge and birth preparedness between the intervention and the control group after the intervention (*p* < .001). The mean knowleadge score was (M = 9.531,SD = 2.666 in the intervention compared to M = 6.518,SD = 4.304 in the control, equivalent to an effect size of 85% of the intervention. Meanwhile, the mean score for IBPACR was M = 4.165,SD = 1.365 for the intervention compared to M = 2.631,SD = 1.775 in the control group with an effect size of 90% A multivariate linear regression showed a positive association between the intervention (*p* < 0.001) and level of knowledge (B = 2.910,95%CI = 2.199–3.621) and birth preparediness (B = 1.463,95%CI = 1.185–1.740).

**Conclusion:**

The Interactive mobile messaging alert system demonstrated to be effective in increasing women’s knowledge on danger signs and improving their birth preparedness practices.

## Plain English summary

Despite of a number of global and national efforts to improve women’s health, the death of women during pregnancy, childbirth and after childbirth remains unresolved challenge in many low income countries.

Birth preparedness is a strategy to promote timely use of skilled maternal care. Pregnant women and their families often ignore early warning signs due to lack of adequate information about the key danger signs therefore delay in seeking care. This study therefore tested the effectiveness of the interactive messaging alert system on improving knowledge level on danger signs and birth preparedness practices among pregnant women in Dodoma Municipal.

With the interactive system women were able to receive health education messages on key danger signs and what to prepare for birth and were also able to send and receive individualized responses on a need basis, so it was a two way communication. Four hundred fifty pregnant women were randomly selected for the study, among them 150 were given the intervention and 300 continued with the health education given in the local clinics and saved as control group.

The level of knowledge on key danger signs in the post test was found to be high (77.3%) in the intervention group compared to the control group (48.0%) and the participants in the intervention group were more prepared for birth (70.7%) against 29.7% in the control group.

The Interactive messaging alert used in this study demonstrated to be effective in empowering pregnant women through greater access to information on key danger signs and birth preparedness.

## Background

Despite of a number of global and national efforts to improve women’s health, the death of women during pregnancy, childbirth and after childbirth remains an unresolved challenge in many developing countries, including Tanzania [1]. Almost two decades since the initiation of the Safe Motherhood Initiative, maternal mortality is still soaring high in many low income countries. About 830 women die from pregnancy or childbirth related complications around the world every day [2].

The estimated MMR in the 2015–16 Tanzania Demographic and Health Survey (TDHS- MIS) report was 556/100000 live births which is higher compared to the 2010 TDHS report which was 454/100000 live births [3].

In most developing countries, the underlying cause of maternal deaths during pregnancy and postpartum are attributed to three crucial delays. These include i) identifying life threatening event/danger signs and making the decision to go to the health facility ii) delay in reaching the health facility and iii) delay in receiving appropriate and adequate care at the health facility [4] .

Low knowledge about danger signs delays obstetric care seeking behaviors which contributes to high maternal mortality and morbidity worldwide [5]. Birth preparedness is a strategy to promote timely use of skilled maternal care especially during childbirth, based on the theory that preparing for childbirth reduces delays in obtaining this care. The proportion of preparing for birth and its complications has been found to be low in low-resource settings [6].

Most of the maternal deaths result from complications during and following pregnancy and childbirth. Most of these complications develop during pregnancy and can be prevented or treated if managed early and properly [1] .

Furthermore, Tanzania’s referral system has serious challenges including limited number of ambulances, unreliable logistics, communication system and inadequate community based facilitated referral system [7].

Tanzania has adopted different strategies including efforts to promote safe motherhood and improving survival of the under-five as well as reducing the prevailing high maternal mortality (MMR). Similarly, in the process of improving maternal and child health; the Government of Tanzania has declared maternal and child health services to be free in all government health facilities with the aim of ending preventable maternal and newborn deaths and encouraging women to utilize all the required services [7] . Despite these efforts, MMR in Tanzania remain persistently high [3].

As part of the Sustainable Development Goals (SDG), the United Nations set a target to reduce the global maternal mortality ratio to less than 70 per 100,000 live births, between the year 2016 and 2030 with no individual country exceeding an MMR of 140 maternal deaths per 100,000 live births. SDG number 3, Good Health and Well-Being for People, this aims to achieve universal health coverage including access to essential medicines and vaccines [8] .

In order to reduce and end poor pregnancy outcome worldwide, interventions should be targeted for all pregnant women attending antenatal care service and during childbirth. Antenatal care should be carried out with well supported and effective technologies for comprehensive monitoring and prompt communication when complications or risks are detected.

There is few published data on use of text messaging with cell phones (SMS) as a tool to increasing health related knowledge to pregnant women [9]. Little evidence exists regarding the different types of mobile health applications that can be used in low-resource settings as well [10] .

Therefore this study aimed at assessing the effectiveness of an interactive messaging alert system on improving knowledge of obstetric danger signs, improving individual birth preparedness and complication readiness practices among pregnant women in Dodoma Municipal.

## Methods

### Study setting

This study was carried out at Dodoma Municipal for both the intervention and control group from January to November, 2018. Dodoma Municipality is found in Dodoma Urban District. Dodoma Region is one of Tanzania’s 30 administrative regions and the location of the capital city of the country. It lies centrally in the eastern-central part of the country; it is about 300 miles (480 km) in the coast. Dodoma Urban District is one of the seven districts of Dodoma region. According to the 2012 Tanzania national census, the population of Dodoma Urban District was 410,956 and the area of 2576 km^2^ [11] . Dodoma was one of the regions with the highest maternal mortality rates in Tanzania as in 2012, Dodoma ranked the ninth high burdened region with a maternal mortality rate of 512/100,000 live births [12] .

Within the municipal, there are two major public health facilities; the Makole Health Center which serves as the main antenatal care facility and the Dodoma Regional Referral Hospital which is the highest level referral hospital in the region. In this study, an interactive messaging alert system was developed and pregnant women attending antenatal care services at Makole Health Centre and Chamwino Dispensary were followed and received health education messages regarding their pregnancy. The control groups were taken from other facilities offering ANC and delivery services in Dodoma Municipal which were not selected for the intervention.

### Study design

This was a quasi-experimental study design with control (pre and post-test with a control group). The intervention group was enrolled in the interactive messaging alert system and received health education messages on antenatal care as per WHO guidelines, where as the control group received the normal health education being offered in the local ANC clinics.

### Study population

Both the intervention group and control group consisted of pregnant women who began Antenatal care at less than 20 weeks gestation. Controls were matched to the intervention group by age group, education level and gravidity (number of previous pregnancies) .

### Sample size and sampling techniques

The sample size for the Intervention and Control group was obtained by using the formula for comparing two independent samples (Intervention group versus control group), The values for π0 and π1 was taken from a quasi- experiment that was done in Uganda on Maternal health service utilization and newborn care; In which the proportion of women attending four visit or more at baseline was 51% and after intervention it was 63% [13]. Standard normal deviation of 1.96 at 95% confidence interval (CI) with 5% attrition rate. So, the minimum sample size was 142 plus 5% Attrition =150.The ratio of the Intervention group to control was 1:2, so controls were 300. So, the total sample size in this study was 450 pregnant women.

A purposive sampling method was used to select healthcare facilities offering ANC and delivery care services in Dodoma Municipal. Purposeful sampling was employed to ensure that data came from all levels of the maternal services referral system in the region. That way, the regional hospital and the Makole Health Center were included in the study. One out of 8 dispensaries was randomly selected. Systematic random sampling method was used to select participants for both the intervention and control groups. Every third pregnant woman among those who met the criteria in a given day and agreed to participate in the study was selected until the required number of sample was obtained. In each setting, the criteria of less than 20 weeks gestation was utilized and attending ANC visit for the first time.

### Inclusion and exclusion criteria

All pregnant women who started their ANC first visit below 20 weeks, attending ANC and planning to deliver in Dodoma Municipal and who owned phones and consented to participate were considered for the study. Pregnant women who were receiving pregnancy-related health text SMS from other sources through their mobile phones and those who meet the inclusion criteria and refused to participate were excluded from the study.

In this study all pregnant women who reported at the centres had mobile phone,this might be due to the nature of the study site, Dodoma urban where people are more educated and wealthier compared to rural. However the findings of this study should not be generalized in rural areas. More study need to be done in rural areas to come up with precise conclusion.

Generally in Tanzania percentage of Phone ownership grows exponentially at the rate of 118%, it is therefore not uncommon to find most women especially in urban areas owning cellphones. According to Tanzania Communications Regulatory Authority, in June 2018 mobile users exceeded 41 million in Tanzania.

### Measurements of variables

Knowledge on obstetric and newborn danger signs was measured with 25 items. Participants were asked to mention spontaneously the key danger signs in the four phases (During pregnancy, labor/delivery, after delivery and danger sign in newborn. One point was given for a correct mentioned item with the maximum score being 25 and lowest score 0 for those who failed to mention any of the key danger sign.

*The key danger signs in the four phases included:*

**Phase 1:** Danger signs during pregnancy (vaginal bleeding, swollen hands/face, severe headache, blurred vision, lower abdominal pain). **Phase 2:** Danger signs during labor/childbirth (severe vaginal bleeding, prolonged labor (> 12 h), convulsions, difficulty in breathing and retained placenta). **Phase 3**: Danger signs during postpartum (severe vaginal bleeding, foul-smelling vaginal discharge, and fever). **Phase 4:** Danger signs in the newborn; pitched cry, difficult feeding (unable to suckle), fits (convulsions), loss of consciousness, hot to touch (hyperthermia), difficult breathing, jaundice, failure to pass urine /stool in the first 24 h [14].

*ii) Individual birth preparedness and complications readiness*: This was measured with nine items. The woman was asked to tell important things/supplies that she prepared for birth and for emergencies, and see whether she knew the basic steps of IBPACR i) Knowing expected date of delivery (EDD) which was confirmed in her RCH 4 card, ii) Identified a skilled birth attendant iii) Identified the mode of transport for delivery and/or for obstetric emergency, iv) Saved money v) Identified two blood donors vi) Prepare supplies for birth and emergencies vii) Prepared a person to escort her during labor or in case of emergency viii) Prepared a person to take care of the family in her absence, and ix) Identified health facility for delivery/ or for an emergency. A score of one was awarded for a correct mentioned response and a maximum score was 9 for those who mentioned all the nine basic components of IBPACR and a score of 0 for those who mentioned none.

### Data collection tools and techniques

#### Research instrument/tools

Semi-structured questionnaire (with both closed and open-ended questions) was developed to be interviewer-administered. This ensured that those unable to read and write could fully participate and also to ensure optimal capturing of all the needed information. The questionnaire included questions on socio-demographic characteristics, knowledge of key danger signs during pregnancy, childbirth, postpartum and danger signs in newborn and individual birth preparedness and complication readiness. The questionnaire was first developed in English and then translated later to Kiswahili which is the national language of Tanzania and the language used by the study population. The questionnaire was adopted and modified from Jhpiego and modified to fit the Tanzanian context [15] also from Tanzania Demographic and Health Survey 2015/2016 and from Nepal Demographic and Health Survey [16].

#### The interactive SMS alert system

An interactive messaging alert system was developed and integrated into the computerized database. The application was developed in January, 2018 and moved to a server and connected to a mobile gateway with enhanced capability to handle multiple and simultaneous SMS problems from the system. Specially designed software automatically generated and sent text messages. The information required for the interactive messaging software such as gestational age and mobile phone number were gathered in the first visit and entered into system by the registering nurses with the help of the system administrator. The aim of the SMS components was to provide simple health education information on obstetric and newborn danger signs and information on individual birth preparedness and complication readiness. Text messages were sent to both expecting parents (mother and father). The content of the messages was developed by an inter-professional team of nurse midwives and obstetricians from the College of Health Sciences at the University of Dodoma. The intervention group was pretested with a baseline questionnaire and then started receiving messages. After the intervention they were again given the same questionnaire to ensure consistency as a post-test. The control group was also pre-tested and received the current standard ANC service. They also completed a post-test after delivery.

**Fig. 1:**
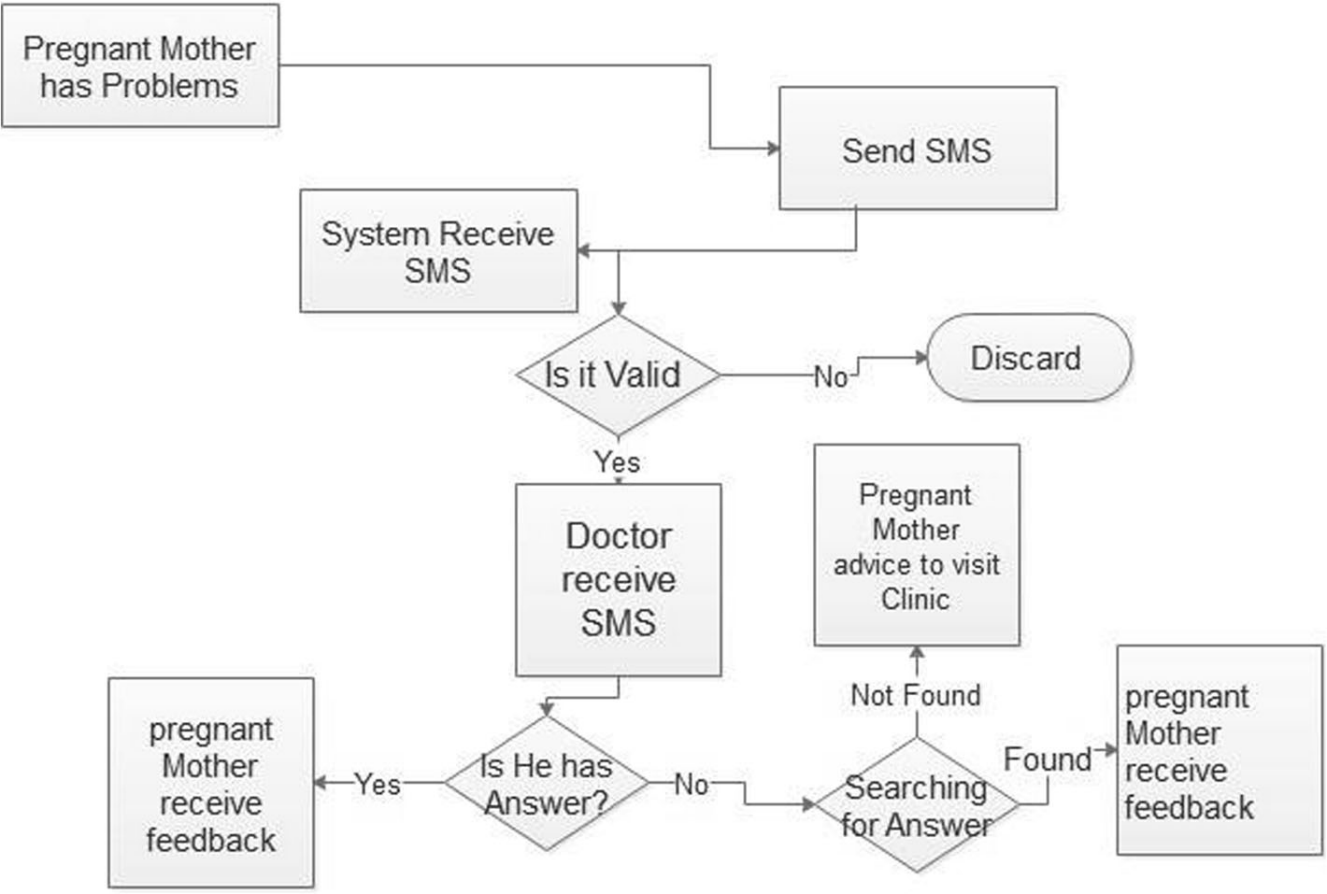
The logical flow diagram which show how this SMS module for two SMS worked

The communication was two-way communication whereby participants were able to send and receive health education messages (see Fig. 1). With the interactive message, a pregnant woman could send text SMS through the system and reach a doctor or nurse. The health provider could respond the message through the system and it was directed back to the pregnant women. The health education SMS messages sent to pregnant women were free of charge for the recipients. The pregnant women were only charged if they called or texted the system in which case, their cost was the standard charge by their individual local network provider. Women were allowed to ask as many questions as they wanted and all the communication were recorded to the system database to identify the patterns of messages and the frequently asked questions. The majority of the participants in the intervention group accessed services and at the end there were more compliments than complaints about the system and appreciations from mothers and their partner.

#### Quality check up of the SMS

Message content was checked for standard and provided as simple SMS in the local language of Swahili. The constructed SMS did not exceed 480 words equivalent to three SMS, sent at different interval of time depending on the gestational age. In the first trimester one message was sent per week, in the second trimester two messages were sent per week and in the third trimester three messages were sent per week. It was done this way to avoid frequently repetition and irritations and to monitor the flow and frequencies of message. The majority of women responded at least once, with 96.5% of pregnant mother texting back for appreciation or to ask questions.

#### The SMS were constructed from the following areas (see Appendix 1)

##### Data processing and analysis

In this study, data was nalyzed by using the Statistical Product for Service Solutions (SPSS) software program version 21. Before conducting the analysis the questionnaires were checked for completeness followed by error checking (data cleaning) by using frequency distribution tables to see if all the data were entered correctly. Both descriptive and inferential analyses were carried out as per the objectives of the study.

Descriptive analysis was used to analyze participant’s characteristics to determine the frequencies and a percentage of their distributions between the two groups. Analysis of covariance (ANCOVA) was used to compare the mean scores between intervention and control group. Simple and multiple linear regression analysis were employed to test the predictors of change in knowledge level and birth preparedness between the intervention and control group adjusting for possible confounders. A confidence interval of 95% with the margin of error 5% (0.05) were used as statistical measure of significance (< 0.05 was regarded as significant while > 0.05 not significant).

### Normality test

Sample characteristics and the type of analysis to be done in this study were determined by running the normality test. Findings showed that parametric methods were the recommended type for data analysis. The visual inspection of their histograms, normal P-P plots showed that the variables were approximately normally distributed. Therefore, the mean, median and range were used as measures of central tendency. The data were also tested for kurtosis and skewness and the data were within the normal range.

## Results

### Social demographic and obstetric characteristics of the participants

A total of 450 pregnant women (*n* = 150 as intervention and *n* = 300 as control) were recruited and participated in the study, with a response rate of 100%. The mean age of respondents in the entire sample was 25.6 ± 6.1 years, with a minimum age of 16 years and maximum age of 48 years. More than half of the study participant, 58.7% (*n* = 264) in the entire sample had primary school education level and majority were married 78.4% (*n* = 78.4%).This study also explored the Obstetric characteristics among the study participants, out of 450 participants 72.4% (*n* = 326) had two to four pregnancies and only 45.3%(*n* = 192) started their first Antenatal visit between 1 and 12 weeks as recommended. The minimum age of the respondent being pregnant at first was 14 years and maximum was 38 years with their mean age and standard deviation being 20 years (3.4) other results are as shown in (Table 1).

**Table 1:** Social demographic characteristics of the participants (*N* = 450)

Variable	Intervention		Control		Total/out of 450	
	n	%	n	%	n	%
Age						
< 20 yrs	21	14.0	61	20.3	82	18.2
20–34 yrs.	111	74.0	212	70.7	323	71.8
≥ 35 yrs	18	12.0	27	9.0	45	10.0
Education status						
Primary school	82	54.7	182	60.7	264	58.7
Secondary school	52	34.7	95	31.7	147	32.7
College/University	16	10.6	23	7.6	39	8.6
Occupational status						
Non-employed	54	36.0	90	30.0	144	32.0
Self-employed	92	61.3	206	68.7	298	66.2
Employed	4	2.7	4	1.3	8	1.8
Marital status						
Not married	25	16.7	72	24.0	97	21.6
Married	125	83.3	228	76.0	353	78.4
Obstetric characteristics of participants						
Gravidity						
1	33	22.0	64	21.3	97	21.6
2–4	109	72.7	217	72.3	326	72.4
≥ 5	8	5.3	19	6.4	27	6.0
Parity						
1	37	24.7	84	28.0	121	26.9
2–4	107	71.3	202	67.3	309	68.7
≥ 5	6	4.0	14	4.7	20	4.4
Gestation age at delivery in weeks						
< 37 weeks	17	11.3	38	12.7	55	12.2
37–40 weeks	116	77.4	241	80.3	357	79.4
≥ 40 weeks	17	11.3	21	7.0	38	8.4
Gestation age at first visit in weeks						
1–12 weeks	56	37.3	136	45.3	192	42.7
13–20 weeks	94	62.7	164	54.7	258	57.3
Age at first pregnancy in years						
< 20 yrs	58	38.7	156	52.0	214	47.4
20–34 yrs	90	60.0	143	47.7	234	52.0
≥ 35 yrs	2	1.3	1	0.3	3	0.6
Total number of antenatal care visits						
1–3 visits	14	9.3	72	24.0	86	19.1
≥ 4 visits	136	90.7	228	76.0	364	80.9

### Knowledge on obstetric and newborn danger signs

Findings showed that at baseline only 46.0% in the intervention and 44.7% of the participants had knowledge on obstetric and newborn danger signs. Whereas after the intervention was found to be 77.3% in the intervention against 48.0% in the control group.

Normality test was performed on knowledge of obstetric and newborn danger signs scores. Scores appeared to be approximately normally distributed with most scores in the histogram occurring at the center. The mean score was 7.5, std. =3.815, *N* = 450, maximum score = 17 minimum = 0, Range 17, Skewness = 0.513 and kurtosis = 1.013. This was also supported by the normal P-P plot where by most of the scores were drawn in the straight line.

### The mean change in knowledge on obstetric and newborn danger signs between intervention and controls

Analysis of covariance (Ancova) between groups (intervention and control) was conducted to compare the mean score differences while statistically controlling for covariates that could influence scores on the dependent variable and hence increase the power and sensitivity of the test. As shown in Table 2 below, At baseline there were no significant differences on mean scores between the intervention and control group on knowledge level. After the intervention there was significant differences between the two groups on the means scores on knowledge level on obstetric and newborn danger signs (M = 9.531, SD = 2.666) for intervention and (M = 6.518,SD = 4.304) for control and a *p* < 0.001 at 95% confidence interval. The Cohen’s d indicated the effect size of the intervention to be 85% which is the large effect size.

**Table 2:** The mean score change on danger signs between intervention and controls (N = 450)

Variables	Control		Intervention		*P*-value	Cohen’s d effect size
	Mean score (SD)	95% CI (lower –upper)	Mean score (SD)	95% CI (lower –upper)		
Knowledge						
Pre-test	4.71 (3.382)	4.344–5.235	4.78 (3.227)	4.303–5.495	0.826	
Post-test	6.518 (4.304)	6.145–6.891	9.531(2.666)	9.003–10.059	0.000	0.85

*Ancova test results

### Predictors of knowledge on obstetric and newborn danger signs between the two groups

Linear regressions were also performed to assess the effect of intervention on the level of knowledge on danger signs after controlling the influence of other factors such as age, education level, parity and gestation age at first visit. In this model only the intervention (*p* < 0.001) and education level at college/university (p < 0.001) were associated with high level of knowledge (B = 2.910, 95%CI = 2.199–3.621) and (B = 1.330, 95%CI = 0.144–2.516) as compared to that in the control group with *p* = 0.245. Results for other predictors are as shown in Table 3 below.

**Table 3:** Multivariate Linear Regression for Predictors of level of knowledge (*N* = 45)

Variables	B	95% CI lower – upper	P-value
Constant	1.149	−0.791-3.089	0.245
Intervention	2.910	2.199–3.621	< 0.001
Age of the participants in years	0.015	−0.055-085	0.672
College/University education	1.330	0.144–2.516	0.028
Gestation age at first visit in weeks	− 0.024	−0.125-0.077	0.642
Parity	0.234	0.146–0.615	0.227

### Practices of individual birth preparedness and complication readiness practices

Baseline and post-test was given in both the intervention and control groups. Participants who reported to be prepared for birth and complication at baseline were only 20.0% in the intervention group and 17.0% in the control group and after the intervention were found to be 70.7% in the intervention against 29.7% in the control group.

Normality test was performed on IBPACR scores, where by scores appear to be approximately normally distributed with most scores in the histogram occurring at the center. The mean score = 3.14, Std = 1.802, maximum score = 9, minimum score = 0, range = 9, *N* = 450,Skewness =0.432 and kurtosis = 0.181.This was also supported by the normal P-P plot where by most of the scores were drawn in the straight line .

### The mean change on individual birth preparedness practices

Analysis of covariance between groups (intervention and control) was conducted to compare the mean score differences while statistically controlling for covariates that could influence scores on the dependent variable and hence increase the power and sensitivity of the test. At baseline there were no significant differences on mean scores between the intervention and control group on IBPACR scores *p* = 0.887. After the intervention, participants on the Intervention group were more prepared for birth with the mean and standard deviation scores (M = 4.17, SD = 1.365) compared to control group (M = 2.63, SD = 1.775) with a *p* < 0.001 at 95% confidence interval. The Cohen’s d indicated the effect size of the intervention to be 90% which is also the large size (Table 4).

**Table 4:** The mean change on birth preparedness practices

Variables	Control		Intervention		P-value	Cohen’s d effect size
	Mean score (SD)	95% CI (lower –upper)	Mean score (SD)	95% CI (lower –upper)		
IBPACR						
Pre-test	1.95(1.205)	1.736–2.034	1.97(1.102)	1.706–2.264	0.887	
Post-test	2.631 (1.775)	2.474–2.788	4.165 (1.365)	3.942–4.387	0.000	0.9

*Ancova test results

### Predictors of individual birth preparedness practices

Linear regressions were also performed to assess the association of the intervention on predicting the change on IBPACR after controlling the influence of other factors such as age, education level and gestational age at first visit. In this modal, the intervention (p < 0.001) and education at college/University level (p < 0.001) were associated with high IBPACR (1.463, 95% CI = 1.185–1.740) and (1.034, 95%CI = 0.578–1.490) other factors did not show statistically significant association (Table 5).

**Table 5:** Predictors of Individual Birth Preparedness (*N* = 450)

Variable	B	95% CI lower – upper	P- value
Constant	0.095	− 0.651-0840	0.803
Intervention	1.463	1.185–1.740	< 0.001
Age of the participants in years	0.020	−0.001-0.042	0.059
College/University education	1.034	0.578–1.490	< 0.001
Gestation age at first visit in weeks	0.000	−0.040-0.963	0.984

## Discussion

Through this study, the effectiveness of interactive messaging alert system was explored and its effectiveness on improving knowledge level on obstetric and newborn danger signs, individual birth preparedness and complication readiness was determined.

### The effect of interactive messaging alert system on knowledge of obstetric and newborn danger signs

The overall level of knowledge on obstetric and newborn danger signs in the post test was found to be high (77.3%) in the intervention group compared to (48.0%) in the control group in the current study. These finding were quite different with the study that was done in Chamwino District on knowledge of obstetric danger signs on recently delivered women,which revealed only 25.2% of respondent to be knowledgeable [14]. These differences between the control and the intervention group might be due to the effect of IMAS of which pregnant women in the intervention group had frequent health education messages and more interaction with the health care giver compared to the controls. On top of that the results of the current were different as compared to those of Chamwino probably due to the study methodologies including the intervention itself and the study site as the current study took place in urban where the services are more improved compared to rural areas.

Findings from this study suggest that IMAS had a positive effect on improving the level of knowledge on obstetric and newborn danger signs. The participants in the intervention group could highly improve the knowledge as compared to the control group. This finding is supported by a Canadian study on text messaging for prenatal education [17] which found its effectiveness to be 70% in providing prenatal education.

Other factors, such as education level, also had a significant effect and should be addressed. Participants with college or university education were more likely to improve their level of knowledge on obstetric and newborn danger signs compared to those with primary level. This might be due to their high ability to understand and interpret the text messages sent to them as it has been seen in other studies in high resource settings [18].

To determine the difference in mean score on knowledge level between the two groups at baseline and in the post test, Analysis of covariance (Ancova) was done. The post-test mean scores indicated that level of knowledge improved more in the intervention group as compared to the control group with the effect size of the intervention being 85%. These significant changes were interpreted and referred to be due to the effect of the IMAS employed in the intervention group.

In addition to that, linear regressions on the effect of interactive messages on knowledge level indicated the positive effect and unique contribution of the intervention in influencing the level of knowledge on obstetric and newborn danger sign in the intervention group. Every time the intervention was applied, knowledge level increased by three times as compared to the control group after controlling the effect of other predictors. This findings correlates with the study on SMS messaging to be an effective tool for reaching pregnant women with health information [9] .

### Individual birth preparedness and complication readiness practices

Preparing for childbirth and being ready for obstetric complications influences action in the event of obstetric emergencies and reduces delay in accessing care. In this study the overall individual preparedness for birth and complication readiness in the post test was found to be 70.7% in the intervention against 29.7% in the control group.

The findings in the intervention group differ with the findings from the study on birth preparedness practice and complication readiness that was done in Ethiopia in which only 48.5% of the participants were prepared [19]. The differences between intervention and control group and the study in Ethiopia could be attributed by the effect of health education messages sent to the intervention group which proves the system to be effective and the methodology used in this study. The most known components were saving money and preparing important supplies such as clothes. Findings from this study suggest that both IMAS and having a higher educational level had a significant effect on IBPACR. The participants in the intervention group were more prepared compared to the control group. Participants with college or university level education were more likely to be prepared as compared to those with lower level. This might be due to their high ability to understand and interpret the text messages sent to them. Also educated women would be more inclined to understand the importance of a birth plan, to adhere to the counseling provided at ANC and may also have more health decision-making power. Finding above link with those found on the Text4baby program done in Atlanta that observed that women who were less educated had lower health literacy and were more likely to have interrupted messages [20].

To compare the mean scores differences pre and post-test between groups (Ancova analysis) was done and revealed that IMAS could promote IBPACR better than depending only on the routine ANC visit. This was evident as participants in the intervention group looks more prepared when asked as compared to control group. The mean scores in the intervention group were higher compared to that in the control group with the intervention effect size being 90%.There was also a significant change in mean scores within the group at baseline and after the intervention.

Furthermore, linear regressions on the effect of interactive messages on IBPACR found a positive effect in which every time the intervention was applied there was an increase by 1.5 IBPACR in the intervention groups compared to the control group after controlling the effect of other factors such as age and education level.

Based on the above findings the researchers concluded Interactive mobile messaging system can enhance IBPACR practices better than the conventional antenatal clinics hence reducing the effect caused by the second and first delays on maternal morbidity and mortality.

### Recommendation

More study are needed to test the efficacy of this intervention in rural areas in order to reach a precise conclusion. This is particular important for scalling up the intervention to other setting in the country and the region at large.

## Conclusion

The study findings have revealed that an interactive mobile messaging alert system yields better outcome to participants in the part of health education as compared to conventional antenatal care health education provided in local ANC clinics. Levels of knowledge, birth preparedness and were higher in the intervention group compared to the control group. Educational level was also a significant predicator for individual birth preparedness, complication readiness, level of knowledge on obstetric and newborn danger signs, so it should also be addressed for better outcome of this intervention.

The high use and feedback given by the participants about the system and the messages sent to them, demonstrates that women are eager to learn more about maternal health beyond what is provided in the standard antenatal clinics. By providing pregnant women with a mechanism for accessing timely evidenced-based health information, the interactive message alert system has contributed in raising awareness on key danger signs and preparing for birth and emergencies for women in the study. The use of SMS technology to disseminate health information is a promising approach in our settings to improve monitoring of pregnant women and increase maternal health care service utilization.

## Data Availability

The datasets used and analyzed in the current study are available from the corresponding author on reasonable request.

## Appendix

The SMS were constructed from the following areas:

i. ***Individual birth preparedness and complication readiness;*** *Pregnant women were reminded on Knowing their expected date of delivery, prepare a skilled birth attendant and a health facility and prepare at least two blood donors in case of emergency. They were also texted to identify a person who will take care of the family in their absence and a person who will escort them to the hospital, arrange for transportation, save money and prepare important supplies which are needed during childbirth and during emergency.*
i.) **ii*****) SMS on knowledge of danger signs during pregnancy;*** *Covered the following signs: Vaginal bleeding, loss of consciousness, severe headache and dizziness, severe vomiting of more than 3 times per day and leaking fluid from the vagina*. S*evere abdominal pain decreased or absent fetal movements, swollen hands, legs, ankles and face, or reduced or no fetal movements. Contractions or labor pain before completed 37 weeks, difficulty in breathing and blurred vision*.
ii.) **iii)** ***SMS on knowledge of danger signs during labor and childbirth;*** *Covered the following signs: vaginal bleeding, severe headache, loss of consciousness and convulsions and rupture of the membrane in the early stage of labor. Labor lasting more than 12 h, fever and difficult breathing, Fatigue and blurred vision.*
iii.) ***iv) SMS on knowledge of danger signs after childbirth;*** *Included the following signs: Abnormal vaginal bleeding, placenta not delivered within one hour after delivery, blurred vision and loss of consciousness or convulsions. Pain in abdomen, breast or nipple pain, fever and difficult breathing and foul smell vaginal discharge. Pain around vagina, being unable to breastfeed and fatigue.*
iv.) ***v) SMS on knowledge of danger signs in newborns;*** *Covered the following signs: High pitched cry, being unable to suck, blueness of lips, tongue or hands, and fits or loss of consciousness. Unable to pass urine and stool or both within 24 h after delivery, fever, weight below 2.5 kg and bleeding from the umbilical cord. Difficult breathing, irritability, being born prematurely and cold to touch*

## Abbreviations

ANC: Antenatal care
BEMOC: Basic Emergency Obstetric Care
CR: Complication Readiness
EMOC: Emergency Obstetric Care
FANC: Focused Antenatal Care
IBPACR: Individual Birth Preparedness and Complication Readiness
IMAS: Interactive Messaging Alert System
MMR: Maternal Mortality Ratio
MoHCDGEC: Ministry of Health Community Development Gender, Elderly and Children
NBS: Nation Bureau of Statistics
SMS: Short Message Service
TDHS-MIS: Tanzania Demographic and Health Survey-Malaria Indicator Survey
UDOM: University of Dodoma
WHO: World Health Organization
